# Data block decomposition and intelligent secure acquisition of microdata

**DOI:** 10.1038/s41598-023-32328-7

**Published:** 2023-04-04

**Authors:** Xiuquan Zhang, Lin Shen, Kaiquan Shi

**Affiliations:** 1 School of Mathematics and Statistics, Huanghuai University, Zhumadian, China; 2 School of Mathematics and Systems Science, Shandong University, Jinan, China

**Keywords:** Applied mathematics, Information technology

## Abstract

P-sets (P stands for Packet) is a set model with dynamic characteristics, which is obtained by introducing dynamic characteristics into Cantor set and improving Cantor set. According to the fact that the characteristics of class I big data are completely consistent with the basic characteristics of P-sets, this paper gives research on theory and application on class I big data from the view of mathematics. Here we introduce Class I big data which need some new definitions of data block, microdata and data link. Based on these concepts, decomposition theorem of data block and microdata relation theorem are given, and then attribute reasoning theorem and microdata intelligent discovery and the intelligent secure acquisition algorithm of microdata are also proposed. By using these theoretical results, the applications of secure acquisition of microdata are presented. In summary, P-sets mathematical model provides a new theory and method for studying class I big data.

## Introduction

Big data is a new and independent field formed by the mutual penetration and intersection of mathematics, statistics, probability theory, computer technology and humanities. As applications, big data and its attributes are interdependent, and the dynamic changes of big data keep pace with those of its attribute set, which is the dynamic nature of big data. Big data and its sub-data have dynamic characteristics. The attribute $\alpha_{i}$ of data element $x_{i}$ satisfies the "conjunctive normal form" or "disjunctive normal form" in mathematical logic. The attribute $\alpha_{i}$ of class I big data element $x_{i}$ meets the characteristic of attribute "conjunction"; meanwhile, the attribute $\alpha_{j}$ of class II big data element $x_{j}$ meets the attribute "disjunctive" characteristic. Based on these, we divide big data into two types: I and II. Dynamic and attribute characteristics are their common characteristics. Class I big data $(x)$ has the following characteristics:

1. $(x)$ has dynamic characteristics, some data elements $x_{i}$ are deleted from $(x)$, and $(x)$ becomes $(x)^{{\overline{F} }}$; whereas other data elements $x_{j}$ outside $(x)$ are supplemented from outside $(x)$ to inside $(x)$,$(x)$ becomes $(x)^{F}$;$(x)^{{\overline{F} }}$ and $(x)^{F}$ together constitute big data $(x)$, where $(x)^{{\overline{F}}} \subseteq (x)$ and $(x) \subseteq (x)^{F}$;
2. The attribute set $\alpha$ of $(x)$ satisfies "attribute conjunction" characteristic, the concept of "conjunction" comes from mathematical logic;
3. Data element $x_{j}$ coexists with its attribute $\alpha$;
4. Big data $(x)$ has an important relationship with mathematical concepts and mathematical models.

Characteristics 1–4 are hidden in big data, which cannot be lost or avoided.

Many scholars have devoted themselves to the application research of combining mathematical models with big data, and have achieved some achievements. Based on the mechanism analysis of big data technology in the food supply chain. Li et al.^1^ established a basic mathematical model called PEFS to evaluate and optimize the stability of the food system. A prediction model was estimated and evaluated using vector autoregressive model with time series data of long- and short-term interest rates^2^. Based on big data, Fan et al.^3^ proposed a grey predictive mathematical model which used to analysis on the effect of Escherichia coli infection on patients with lupus nephritis. However, there is little research on big data features 1–4 in these references, and they focus on combining big data with other disciplines and only give application research, but ignore the theoretical research of big data, pay no attention to the mathematical concept connotation and dynamic characteristics contained in big data, and no one combines big data with mathematics to give theoretical research of big data from the perspective of mathematics.

Shi^4^ obtained P-sets by introducing dynamic characteristics into Cantor set $X$ and improving $X$. Cantor set $X$ is given, and $\alpha$ is attribute set of $X$, the concepts and characteristics of P-sets are as follows:

I Supplementing some attributes in $\alpha$, then $\alpha$ becomes $\alpha^{F}$, $\alpha \subseteq \alpha^{F}$;$X$ becomes internal P-sets $X^{{\overline{F} }}$, $X^{{\overline{F} }} \subseteq X$;
II Deleting some attributes in $\alpha$, then $\alpha$ becomes $\alpha^{{\overline{F} }}$, $\alpha^{{\overline{F} }} \subseteq \alpha$; $X$ becomes outer P-sets $X^{F}$, $X \subseteq X^{F}$;
III If attributes are supplemented and deleted in $\alpha$ at the same time,$\alpha$ becomes $\alpha^{F}$ and $\alpha^{{\overline{F}}}$, where $\alpha^{{\overline{F}}} \subseteq \alpha \subseteq \alpha^{F}$; $X$ becomes internal P-sets $X^{{\overline{F} }}$ and outer P-sets $X^{F}$, where $X^{{\overline{F} }} \subseteq X \subseteq X^{F}$; or $X$ becomes $(X^{{\overline{F} }} ,X^{F} )$, $(X^{{\overline{F} }} ,X^{F} )$ is the P-sets generated by $X$.

The mathematical characteristics of the P-sets such as quantitative characteristics, algebraic characteristics, geometrical characteristics, random characteristics, and theory applications are studied by scholars. By using P-sets, L. A. Zadeh fuzzy set is improved^5^. An algebraic model of P-sets is proposed^6^. By introducing assistant set into function P-sets to expand the function P-sets^7^. More dynamic characteristics and applications of P-sets are discussed^8–10^. P-information fusion and application based on P-sets are obtained^11–14^. The inverse P-sets is proposed^15–21^, which is the dual form of P-sets. Function P-sets is given^22,23^, which is the functional form of P-sets; and the function inverse P-sets is also proposed^24–26^, which is the functional form of inverse P-sets. However, some of these achievements generalize or expand P-sets, while others discuss their applications in dynamic information systems using P-sets as tools, such as intelligent data mining, risk tracking and recognition, and intelligent image recognition. no one has applied the P-sets mathematical model to big data theory and application research in the available literature.

P-sets and class I big data have the same dynamic characteristics and attribute "conjunction" characteristics. In view of I-III, it is easy to get that P-sets is a new mathematical model and method for studying big data. It provides theoretical support for studying the structure, characteristics and basic theory of class I big data.

The purpose of this paper is to give research on theory and application of Class I big data by using the mathematical model with dynamic characteristics.

## Dynamic model with attribute conjunction

Cantor set $X = \{ x_{1} ,x_{2} , \ldots ,x_{q} \} \subset U$ is given, and $\alpha = \{ \alpha_{1} ,\alpha_{2} , \ldots ,\alpha_{k} \} \subset V$ is the attribute set of $X$, $X^{{\overline{F} }}$ is referred to as internal P-sets generated by $X$, referred to as that $X^{{\overline{F} }}$ is internal P-sets for short,

1 $$X^{{\overline{F} }} = X - X^{ - }$$

$X^{ - }$ is referred to as $\overline{F}$-element deleted set of $X$,

2 $$X^{ - } = \{ x_{i} |x_{i} \in X,\mathop f\limits^{\_} (x_{i} ) = u_{i} \overline{ \in }X,\mathop f\limits^{\_} \in \mathop F\limits^{\_} \} .$$

If attribute set $\alpha^{F}$ of $X^{{\overline{F} }}$ meets

3 $$\alpha^{F} = \alpha \cup \{ \alpha_{i}^{\prime } |f(\beta_{i} ) = \alpha_{i}^{\prime } \in \alpha ,f \in F\} .$$

where in (3), $$\beta_{i} \in V,\beta_{i} \overline{ \in }\alpha ,f \in F$$ changes $$\beta_{i}$$ into $$f(\beta_{i} ) = \alpha_{i}^{\prime } \in \alpha$$; in (1), $$X^{{\overline{F} }} \ne \emptyset ,$$ $$X^{{\overline{F} }} = \{ x_{1} ,x_{2} , \ldots ,x_{p} \} ,$$$$p < q;p,q \in N^{ + }$$.

Cantor set $X = \{ x_{1} ,x_{2} , \ldots ,x_{q} \} \subset U$ is given, and $\alpha = \{ \alpha_{1} ,\alpha_{2} , \ldots ,\alpha_{k} \} \subset V$ is the attribute set of $X$, $X^{F}$ is referred to as outer P-sets generated by $X$, referred to as that $X^{F}$ is outer P-sets for short,

4 $$X^{F} = X \cup X^{ + } ,$$

$X^{ + }$ is referred to as $F$-element supplemented set of *X*,

5 $$X^{ + } = \{ u_{i} |u_{i} \in U,u_{i} \overline{ \in }X,f(u_{i} ) = x_{i}^{\prime } \in X,f \in F\} .$$

If attribute set $\alpha^{{\overline{F} }}$ of $X^{F}$ meets

6 $$\alpha^{{\overline{F} }} = \alpha - \{ \beta_{i} |\mathop f\limits^{\_} (\alpha_{i} ) = \beta_{i} \overline{ \in }\alpha ,\mathop f\limits^{\_} \in \mathop F\limits^{\_\_} \} .$$

where in (6), $\alpha_{i} \in \alpha ,\mathop f\limits^{\_} \in \mathop F\limits^{\_\_}$ changes $\alpha_{i}$ into $\overline{f}(\alpha_{i} ) = \beta_{i} \overline{ \in }\alpha$, $\alpha^{{\overline{F} }} \ne \emptyset$; in (4), $X^{F} = \{ x_{1} ,x_{2} , \ldots ,x_{r} \} ,$ $q < r;$ $q,r \in N^{ + }$.

The set pair which is composed of internal packet set $X^{{\overline{F} }}$ and outer packet set $X^{F}$ is referred to as P-sets generated by $X$, referred to as P-sets for short and written as

7 $$(X^{{\overline{F} }} ,X^{F} ).$$

From (9) and (11), we get:

8 $$\{ (X_{i}^{{\overline{F} }} ,X_{j}^{F} )|i \in I,j \in J\}$$

(8) is referred to as the family of P-sets generated by $X$, which is the general expression of P-sets, where both $I$ and $J$ are indicator sets.

From (1) to (8), we get:

### Proposition 1

Under the condition of $F = \overline{F} = \emptyset$, P-sets $(X^{{\overline{F} }} ,X^{F} )$ and Cantor set $X$ meet:

9 $$(X^{{\overline{F} }} ,X^{F} )_{{F = \overline{F} = \emptyset }} = X.$$

### ***Proof***

1. if $F = \overline{F} = \emptyset$, from (3), we get: $\alpha^{F} = \alpha \cup \{ \alpha_{i}^{\prime } |f(\beta_{i} ) = \alpha_{i}^{\prime } \in \alpha ,f \in F\}$.

$= \alpha \cup \emptyset = \alpha$,$\{ \alpha_{i}^{\prime } |f(\beta_{i} ) = \alpha_{i}^{\prime } \in \alpha ,f \in F\} = \emptyset$;and $X^{ - } = \emptyset$ in the Formula (2), $X^{{\overline{F} }} = X - X^{ - } = X - \emptyset = X$ in the Formula (1).

2. if $F = \overline{F} = \emptyset$, from (6), we get: $\alpha^{{\overline{F} }} = \alpha - \{ \beta_{i} |\mathop f\limits^{\_} (\alpha_{i} ) = \beta_{i} \overline{ \in }\alpha ,\mathop f\limits^{\_} \in \mathop F\limits^{\_\_} \}$

$\alpha - \emptyset = \alpha$; $\{ \beta_{i} |\overline{f}(\alpha_{i} ) = \beta_{i} \overline{ \in }\alpha ,\overline{f} \in \overline{F}\} = \emptyset$, and $X^{ + } = \emptyset$ in the Formula (5), $X^{F} = X \cup X^{ + } = X \cup \emptyset = X$ in the Formula (4).

Based on 1 and 2, we can complete this Proposition.

### Proposition 2

Under the condition of $F = \overline{F} = \emptyset$, the family of P-sets $\{ (X_{i}^{{\overline{F} }} ,X_{j}^{F} )|i \in I,j \in J\}$ and Cantor set $X$ meet:

10 $$\{ (X_{i}^{{\overline{F} }} ,X_{j}^{F} )|i \in I,j \in J\}_{{F = \overline{F} = \emptyset }} = X$$

The proof is similar to Proposition 1, and it is omitted.

From Formula (1)–(8) and Proposition 1 and 2, we can easily get the dynamic characteristics of P-sets as follows:

Under the condition we continuously add attributes to $\alpha$, $X$ generates an internal P-sets $X_{i}^{{\overline{F}}}$; similarly we delete attributes continuously in $\alpha$, $X$ dynamically generates an outer P-sets $X_{j}^{F}$; if the attributes are supplemented and deleted at the same time in $\alpha$, $X$ dynamically generates P-sets $(X_{i}^{{\overline{F}}} ,X_{j}^{F} )$,$i,j = 1,2, \ldots ,n$.

### *Remark*

1. $U$ is a finite element domain and $V$ is a finite attribute domain;

2. $f \in F$ and $\mathop f\limits^{\_} \in \mathop F\limits^{\_\_}$ are element (attribute) transfer; $F = \{ f_{1} ,f_{2} \ldots f_{n} \}$ and $\mathop F\limits^{\_\_} = \{ \mathop {f_{1} }\limits^{\_} ,\mathop {f_{2} }\limits^{\_} , \ldots ,\mathop {f_{n} }\limits^{\_} \}$ are the family of element (attribute) transfer, element (attribute) transfer is a concept of function or transformation;

3. The characteristics of $f \in F$ are that: for element $u_{i} \in U,u_{i} \overline{ \in }X,f \in F$ changes $u_{i}$ into $f(u_{i} ) = x_{i}^{\prime } \in X$; for attribute $\beta_{i} \in V,\beta_{i} \overline{ \in }\alpha ,f \in F$ changes $\beta_{i}$ into $f(\beta_{i} ) = \alpha_{i}^{\prime } \in \alpha$;

4. The characteristics of $\mathop f\limits^{\_} \in \mathop F\limits^{\_\_}$ are that: for element $x_{i} \in X$, $\mathop f\limits^{\_} \in \mathop F\limits^{\_\_}$ changes $x_{i}$ into $\overline{f}(x_{i} ) = u_{i} \overline{ \in }X$; for attribute $\alpha_{i} \in \alpha ,$ $\mathop f\limits^{\_} \in \mathop F\limits^{\_\_}$ changes $\alpha_{i}$ into $\overline{f}(\alpha_{i} ) = \beta_{i} \overline{ \in }\alpha$;

5. The dynamic characteristics of Formula (1) are the same as the dynamic characteristics of down-counter $T = T - 1$;

6. The dynamic characteristics of Formula (4) are the same as the dynamic characteristics of accumulator $T = T + 1$. For example, for the Formula (4) $X_{1}^{F} = X \cup X_{1}^{ + }$, let $X = X_{1}^{F}$, $X_{2}^{F} = X \cup X_{2}^{ + } = (X_{{1}}^{F} \cup X_{1}^{ + } ) \cup X_{2}^{ + } , \cdot \cdot \cdot$, so on.

### Fact and evidence of existence of P-sets

$X = \{ x_{1} ,x_{2} ,x_{3} ,x_{4} ,x_{5} \}$ is a finite commodity element set of five apples, and $\alpha = \{ \alpha_{{1}} ,\alpha_{{2}} ,\alpha_{{3}} \}$ is the attribute set confined in $X$, where $\alpha_{1}$ denotes red color, $\alpha_{2}$ denotes sweet taste, $\alpha_{3}$ denotes produced by Henan province of China. Obviously, $x_{i}$ has attributes $\alpha_{1} ,\alpha_{2}$ and $\alpha_{3}$; the attribute $\alpha_{i}$ of $x_{i}$ meets “conjunctive normal form”, $\forall x_{i} \in X,i = 1,2, \cdot \cdot \cdot ,5$, moreover

$$\alpha_{i} = \alpha_{1} \wedge \alpha_{2} \wedge \alpha_{3}$$

Let $\alpha_{4}$ denotes weight is 150 g, supplementing attribute $\alpha_{4}$ in $\alpha$, $\alpha$ is changed into $\alpha^{F} = \{ \alpha_{{1}} ,\alpha_{{2}} {,}\alpha_{{3}} \} \cup \{ \alpha_{{4}} \}$, and $X$ is changed into internal P-sets $X^{{\overline{F}}} = X - \{ x_{4} ,x_{5} \} = \{ x_{1} ,x_{2} ,x_{3} \}$. Obviously,$x_{i}$ has attributes $\alpha_{1} ,\alpha_{2} ,\alpha_{3}$ and $\alpha_{4}$, $\forall x_{i} \in X^{{\overline{F}}} ,i = 1,2,5$, moreover

$$\alpha_{i} = (\alpha_{1} \wedge \alpha_{2} \wedge \alpha_{3} ) \wedge \alpha_{4} = \alpha_{1} \wedge \alpha_{2} \wedge \alpha_{3} \wedge \alpha_{4} .$$

If deleting attribute $\alpha_{3}$ in $\alpha$, $\alpha$ is changed into $\alpha^{{\overline{F} }} = \{ \alpha_{1} ,\alpha_{2} ,\alpha_{3} \} - \{ \alpha_{3} \} = \{ \alpha_{1} ,\alpha_{2} \}$, and $X$ is changed into outer P-sets $X^{F} = X \cup \{ x_{6} ,x_{7} \} = \{ x_{1} ,x_{2} ,x_{3} ,x_{4} ,x_{5} ,x_{6} ,x_{7} \}$. Obviously,$x_{i}$ has attributes $\alpha_{1} ,\alpha_{2}$. $\forall x_{i} \in X^{F} ,$ $i = 1,2, \cdot \cdot \cdot ,7$, moreover

$$\alpha_{i} = (\alpha_{1} \wedge \alpha_{2} \wedge \alpha_{3} ) - \wedge \alpha_{3} = \alpha_{1} \wedge \alpha_{2}$$

**Figure 1:**
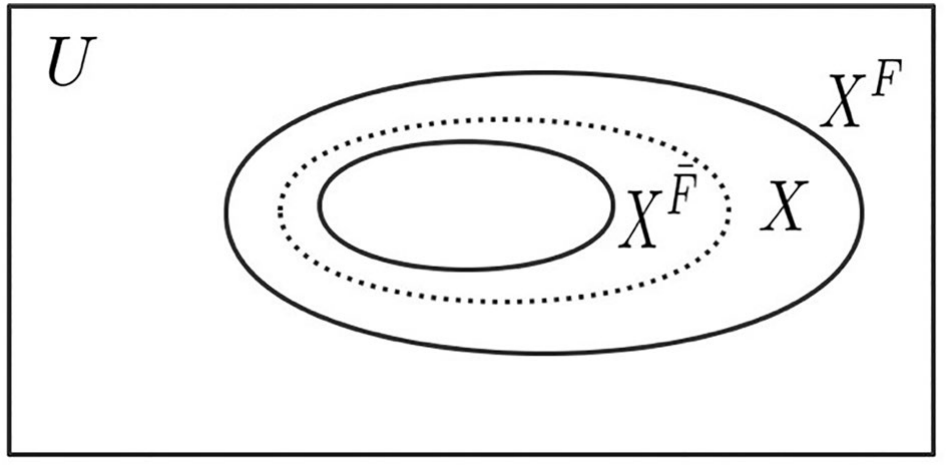
$X^{{\overline{F}}}$ and $X^{F}$ are the internal P-sets and outer P-sets generated by $X$ respectively, $X^{{\overline{F}}}$ and $X^{F}$ constitute P-Sets $(X^{{\overline{F}}} ,X^{F} )$;$X^{{\overline{F}}}$ and $X^{F}$ are represented by solid lines, and finite ordinary element set $X$ is represented by dotted lines, $U$ is a finite element domain.

This simple fact and logical feature can be accepted by ordinary people. The relationship among $X^{{\overline{F}}}$, $X^{F}$ and finite ordinary element set $X$ are shown in Fig. 1.

Agreement: we call that $(x) = X,(x)^{{\overline{F} }} = X^{{\overline{F}}}$, $(x)^{F} = X^{F} ,((x)^{{\overline{F}}} ,(x)^{F} ) = (X^{{\overline{F}}} ,X^{F} )$; in "Dynamic model with attribute conjunction" section; $(x),(x)^{{\overline{F} }} ,(x)^{F}$ and $((x)^{{\overline{F} }} ,(x)^{F} )$ are data, element $x_{i}$ in $X$ is data element of $X$, which will be used directly in "Data block decomposition and microdata generation" section–"Microdata intelligent-security acquisition algorithm" section.

By using characteristics and concept of P-sets in "Dynamic model with attribute conjunction" section, we get "Data block decomposition and microdata generation" section.

## Data block decomposition and microdata generation

### Definition 1

If $(x)^{{\overline{F} }}$ and $(x)^{F}$ satisfy.

11 $$(x)^{{\overline{F} }} = \{ (x)_{i}^{{\overline{F} }} |(x)_{i + 1}^{{\overline{F} }} \subseteq (x)_{i}^{{\overline{F} }} ,\alpha_{i}^{F} \subseteq \alpha_{i + 1}^{F} ;i = 1,2,\ldots,n\}$$

12 $$(x)^{F} = \{ (x)_{j}^{F} |(x)_{j}^{F} \subseteq (x)_{j + 1}^{F} ,\alpha_{j + 1}^{{\overline{F} }} \subseteq \alpha_{j}^{{\overline{F} }} ;j = 1,2,\ldots,n - 1\}$$

then $(x)^{{\overline{F} }}$ and $(x)^{F}$ are called $\alpha^{F}$-data block and $\alpha^{{\overline{F} }}$-data block of $(x)$ respectively. Otherwise, $\alpha_{i}^{F}$ is the attribute set of $(x)_{i}^{{\overline{F} }}$,$(x)_{i}^{{\overline{F} }}$ is the $\alpha^{F}$-block of $(x)^{{\overline{F} }}$;$\alpha_{j}^{{\overline{F} }}$ is the attribute set of $(x)_{j}^{F}$,$(x)_{j}^{F}$ is the $\alpha^{{\overline{F}}}$-block of $(x)^{F}$;where $(x)_{i}^{{\overline{F} }} \in (x)^{{\overline{F} }} ,(x)_{j}^{F} \in (x)^{F}$.

### Definition 2

We call $(x)$ is big data composed of $\alpha^{F}$-data block $(x)^{{\overline{F} }}$ and $\alpha^{{\overline{F}}}$-data block $(x)^{F}$ respectively, and.

13 $$\begin{aligned} (x) & = (x)^{{\overline{F}}} \cup (x)^{F} \\ & = (\mathop \cup \limits_{i = 1}^{n} (x)_{i}^{{\overline{F}}} ) \cup (\mathop \cup \limits_{j = 1}^{n} (x)_{j}^{F} ) \\ \end{aligned}$$

### Definition 3

Under the condition of supplementing attributes in $\alpha_{k}^{F}$, if $\nabla (x)_{{k{ + }1}}^{{\overline{F} }} ,(x)_{k}^{{\overline{F}}}$ and $(x)^{ - }$ satisfy.

14 $$\nabla (x)_{{k{ + }1}}^{{\overline{F} }} = (x)_{k}^{{\overline{F} }} - (x)^{ - }$$

then $\nabla (x)_{k}^{{\overline{F} }}$ is called $\alpha_{k}^{F}$-microdata generated by $(x)_{k}^{{\overline{F} }}$, where $(x)^{ - }$ is composed of deleted data elements $x_{i}$ in $(x)_{k}^{{\overline{F} }}$; $\nabla (x)_{{k{ + }1}}^{{\overline{F} }} \subseteq (x)_{k}^{{\overline{F} }}$.

### Definition 4

Under the condition of deleted attributes in $\alpha_{k}^{{\overline{F} }}$, if $\Delta (x)_{k + 1}^{F} ,(x)_{k}^{F}$ and $(x)^{ + }$ satisfy.

15 $$\Delta (x)_{k + 1}^{F} = (x)_{k}^{F} \cup (x)^{ + }$$

then $\Delta (x)_{k + 1}^{F}$ is called $\alpha_{k}^{{\overline{F}}}$-microdata generated by $(x)_{k}^{F}$, where $(x)^{ + }$ is composed of data element $x_{j}$ supplemented into $(x)_{k}^{F}$; where $\Delta (x)_{k + 1}^{F} \supseteq (x)_{k}^{F}$.

From (11) to (15), we get:

### Theorem 1

(Decomposition Theorem of internal $\alpha^{F}$-data block).

The sufficient and necessary conditions for $(x)_{k}^{{\overline{F}}}$ is the $\alpha^{F}$-data block decomposed from $(x)$ are: there is $\Delta \alpha \ne \emptyset$, so that the attribute set $\alpha_{k}^{F}$ of $(x)_{k}^{{\overline{F} }}$ and attribute sets $\alpha$ of $(x)$ satisfy

16 $$\alpha_{k}^{F} - (\alpha \cup \Delta \alpha ) = \emptyset$$

where $$\Delta \alpha \cap \alpha = \emptyset$$.

### ***Proof***

From (1)–(3) in "Dynamic model with attribute conjunction" section, we get:

1. if attribute set $\Delta \alpha$ is supplemented by attribute set $\alpha_{k}^{F}$ of $(x)_{k}^{{\overline{F} }}$ and attribute set $\alpha$ of $(x)$, and it satisfies $\alpha_{k}^{F} = \alpha \cup \Delta \alpha$, or $\alpha_{k}^{F} - (\alpha \cup \Delta \alpha ) = \emptyset$, then $(x)_{k}^{{\overline{F} }}$ is the internal decomposition of $(x)$,$(x)_{k}^{{\overline{F} }} \subseteq (x)$.

2. If $(x)_{k}^{{\overline{F} }}$ is the internal decomposition of $(x)$, then $\alpha_{k}^{F} = \alpha \cup \Delta \alpha$, or $\alpha_{k}^{F} - (\alpha \cup \Delta \alpha ) = \emptyset$, Among them, $\Delta \alpha$ is the attribute set added to $\alpha$,$\Delta \alpha \ne \emptyset$. Theorem 1 is proved from 1 and 2. □

### Theorem 2

(Decomposition Theorem outer $\alpha^{{\overline{F}}}$-data block).

The sufficient and necessary conditions for $(x)_{k}^{F}$ is the $\alpha^{{\overline{F}}}$-data block decomposed from $(x)$ are: there is $\nabla \alpha \ne \emptyset$, so that the attribute set $\alpha_{k}^{{\overline{F} }}$ of $(x)_{k}^{F}$ and attribute set $\alpha$ of $(x)$ satisfy

17 $$\alpha_{k}^{{\overline{F} }} - (\alpha - \nabla \alpha ) = \emptyset$$

where $$\nabla \alpha \cap \alpha \ne \emptyset$$.

Its proof is similar to Theorem 1, and it is omitted.

### Theorem 3

The necessary and sufficient conditions for the existence of $\alpha_{k + 1}^{F}$-microdata $\nabla (x)_{k + 1}^{{\overline{F} }}$ are: the attribute set $\alpha_{k}^{F}$ of $(x)_{k}^{{\overline{F} }}$ and the attribute set $\alpha_{k + 1}^{F}$ of $\nabla (x)_{k + 1}^{{\overline{F} }}$ satisfy.

18 $$card(\alpha_{k + 1}^{F} ) - card(\alpha_{k}^{F} ) > 0$$

### ***Proof***

From Formula (1) to (3) and Formula (14) in "Dynamic model with attribute conjunction" section, we get: $\nabla (x)_{k + 1}^{{\overline{F} }} = (x)_{k}^{{\overline{F} }} - (x)^{ - } = (x)_{k + 1}^{{\overline{F} }}$;if $\alpha_{k}^{F}$ and $\alpha_{k + 1}^{F}$ are attribute sets of $(x)_{k}^{{\overline{F} }}$ and $\nabla (x)_{k + 1}^{{\overline{F} }}$ respectively, then $\alpha_{k}^{F} \subseteq \alpha_{k + 1}^{F}$, or $card(\alpha_{k + 1}^{{\overline{F}}} ) - card(\alpha_{k}^{{\overline{F}}} ) > 0$.□

### Theorem 4

The necessary and sufficient conditions for the existence of $\alpha_{k}^{{\overline{F}}}$-microdata $\Delta (x)_{k + 1}^{F}$ are: the attribute set $\alpha_{k}^{{\overline{F}}}$ of $(x)_{k}^{F}$ and the attribute set $\alpha_{k + 1}^{{\overline{F}}}$ of $\Delta (x)_{k + 1}^{F}$ satisfy.

19 $$card(\alpha_{k + 1}^{{\overline{F}}} ) - card(\alpha_{k}^{{\overline{F}}} ) < 0$$

Its proof is similar to Theorem 3, and it is omitted, where in (18) and (19), $card = cardinal\;number$.

### Theorem 5

($\alpha^{F}$-microdata relation Theorem) If there is $k$, then $\alpha_{k}^{F}$-microdata $\nabla (x)_{k}^{{\overline{F} }}$ meets.

20 $$\mathop \cup \limits_{i = 1}^{k - 1} \nabla (x)_{i}^{{\overline{F} }} \subseteq \nabla (x)_{k}^{{\overline{F} }} \subseteq \mathop \cup \limits_{i = k + 1}^{n} \nabla (x)_{i}^{{\overline{F} }}$$

### Theorem 6

($\alpha^{{\overline{F}}}$-microdata relation Theorem) If there is $\lambda$, then $\alpha_{\lambda }^{{\overline{F}}}$-microdata $\Delta (x)_{\lambda }^{{\overline{F} }}$ meets.

21 $$\mathop \cup \limits_{j = 1}^{\lambda - 1} \Delta (x)_{j}^{F} \subseteq \Delta (x)_{\lambda }^{F} \subseteq \mathop \cup \limits_{j = \lambda + 1}^{n} \Delta (x)_{j}^{F}$$

Propositions 3 and 4 are obtained from (11) to (21):

### Propositions 3

$\alpha^{F}$-data block $(x)_{i}^{{\overline{F} }}$ constitutes $\alpha^{F}$-data block chain of $(x)$ as follows:

22 $$\{ (x)_{n}^{{\overline{F} }} ,\alpha_{n}^{F} \} \leftarrow \{ (x)_{n - 1}^{{\overline{F} }} ,\alpha_{n - 1}^{F} \} \leftarrow \cdots \leftarrow \{ (x)_{2}^{{\overline{F} }} ,\alpha_{2}^{F} \} \leftarrow \{ (x)_{1}^{{\overline{F} }} ,\alpha_{1}^{F} \}$$

### Propositions 4

$\alpha^{{\overline{F} }}$-data block $(x)_{j}^{F}$ constitutes $\alpha^{{\overline{F} }}$-data block chain of $(x)$ as follows:

23 $$\{ (x)_{1}^{F} ,\alpha_{1}^{{\overline{F} }} \} \leftarrow \{ (x)_{2}^{F} ,\alpha_{2}^{{\overline{F} }} \} \leftarrow \cdots \leftarrow \{ (x)_{n - 1}^{F} ,\alpha_{n - 1}^{{\overline{F} }} \} \leftarrow \{ (x)_{n}^{F} ,\alpha_{n}^{{\overline{F} }} \}$$

By utilizing the concepts and the model in "Dynamic model with attribute conjunction" section, and the theoretical results in "Data block decomposition and microdata generation" section, we will give "Attribute reasoning and intelligent discovery of microdata" section.

## Attribute reasoning and intelligent discovery of microdata

Suppose $\nabla (x)_{k + 1}^{{\overline{F} }}$ is the $\alpha_{k}^{F}$-microdata generated by $(x)_{k}^{{\overline{F} }}$, and $\alpha_{k + 1}^{F}$ and $\alpha_{k}^{F}$ are their attribute sets respectively, if they meet

24 $${\text{if}}\;\alpha_{k}^{F} \Rightarrow \alpha_{k + 1}^{F} ,\;{\text{then}}\;\nabla (x)_{k + 1}^{{\overline{F} }} \Rightarrow (x)_{k}^{{\overline{F} }}$$

(24) is referred to as attribute reasoning generated by $\alpha^{F}$-microdata $\nabla (x)_{k + 1}^{{\overline{F} }}$,$\alpha_{k}^{F} \Rightarrow \alpha_{k + 1}^{F}$ is referred to as reasoning condition, $\nabla (x)_{k + 1}^{{\overline{F} }} \Rightarrow (x)_{k}^{{\overline{F} }}$ is referred to as inference conclusion.

Suppose $\Delta (x)_{k + 1}^{F}$ is the $\alpha_{k}^{{\overline{F} }}$-microdata generated by $(x)_{k}^{F}$, and $\alpha_{k + 1}^{{\overline{F} }}$ and $\alpha_{k}^{{\overline{F} }}$ are their attribute sets respectively, if they meet

25 $${\text{if}}\;\alpha_{k + 1}^{{\overline{F}}} \Rightarrow \alpha_{k}^{{\overline{F}}} ,\;{\text{then}}\;(x)_{k}^{F} \Rightarrow \Delta (x)_{k + 1}^{F}$$

(25) is referred to as attribute reasoning generated by $\alpha^{{\overline{F} }}$-microdata $\Delta (x)_{k + 1}^{F}$,$\alpha_{k + 1}^{{\overline{F}}} \Rightarrow \alpha_{k}^{{\overline{F}}}$ is referred to as reasoning condition, $(x)_{k}^{F} \Rightarrow \Delta (x)_{k + 1}^{F}$ is referred to as inference conclusion.where in (24) and (25), “$\Rightarrow$” is equal to“$\subseteq$”.

If $(\nabla (x)_{k + 1}^{F} ,(x)_{k}^{F} )$, $((x)_{k}^{{\overline{F} }} ,\Delta (x)_{k + 1}^{F} )$ and $(\alpha_{k}^{F} ,\alpha_{k + 1}^{{\overline{F} }} )$, $(\alpha_{k + 1}^{F} ,\alpha_{k}^{{\overline{F} }} )$ meet

26 $${\text{if}}\;(\alpha_{k}^{F} ,\alpha_{k + 1}^{{\overline{F} }} ) \Rightarrow (\alpha_{k + 1}^{F} ,\alpha_{k}^{{\overline{F} }} ),\;{\text{then}}\;(\nabla (x)_{k + 1}^{{\overline{F} }} ,(x)_{k}^{F} ) \Rightarrow ((x)_{k}^{F} ,\Delta (x)_{k + 1}^{F} )$$

(26) is referred to as attribute reasoning generated by $(\alpha^{F} ,\alpha^{{\overline{F} }} )$-microdata $(\nabla (x)_{k + 1}^{{\overline{F} }} ,\Delta (x)_{k + 1}^{F} )$,$(\alpha_{k}^{F} ,\alpha_{k + 1}^{{\overline{F} }} ) \Rightarrow (\alpha_{k + 1}^{F} ,\alpha_{k}^{{\overline{F} }} )$ is referred to as reasoning condition, $(\nabla (x)_{k + 1}^{{\overline{F} }} ,(x)_{k}^{F} ) \Rightarrow ((x)_{k}^{F} ,\Delta (x)_{k + 1}^{F} )$ is referred to as inference conclusion. Where (26) represents if $\alpha_{k}^{F} \Rightarrow \alpha_{k + 1}^{F}$, then $\;\nabla (x)_{k + 1}^{{\overline{F} }} \Rightarrow (x)_{k}^{{\overline{F} }}$; if $\alpha_{k + 1}^{{\overline{F} }} \Rightarrow \alpha_{k}^{{\overline{F} }}$, then $\,\,(x)_{k}^{F} \Rightarrow \Delta (x)_{k + 1}^{F}$.

From (24) to (26), we get:

### Theorem 7

($\alpha^{F}$-microdata $\nabla (x)_{k + 1}^{{\overline{F} }}$ intelligent decomposition-discovery Theorem) Under the condition of reasoning (24), $\alpha^{F}$-microdata $\nabla (x)_{k + 1}^{{\overline{F} }}$ is intelligently decomposed—discovered from $\alpha^{{\overline{F} }}$-data block $(x)_{k}^{{\overline{F} }}$, or.

1. $$\nabla (x)_{k + 1}^{{\overline{F} }}$$ and $$(x)_{k}^{{\overline{F} }}$$ satisfy

27 $$\nabla (x)_{k + 1}^{{\overline{F} }} \cap (x)_{k}^{{\overline{F} }} \ne \phi$$

2. The separation coefficient $\eta_{k + 1}^{{\overline{F} }}$ of $\nabla (x)_{k + 1}^{{\overline{F} }}$ separated from $(x)_{k}^{{\overline{F} }}$ satisfies

28 $$\eta_{k + 1}^{{\overline{F} }} - 1 < 0$$

where $\eta_{k + 1}^{{\overline{F} }} = card(\nabla (x)_{k + 1}^{{\overline{F} }} /card((x)_{k}^{{\overline{F} }} )$,$1 = card((x)_{k}^{{\overline{F} }} /card(x)_{k}^{{\overline{F} }} ) = \eta = 1$ is the self separation coefficient of $(x)_{k}^{{\overline{F} }}$.

### ***Proof***

1. From Formula (1)–(3) in "Dynamic model with attribute conjunction" section and Formula (14) in "Data block decomposition and microdata generation" section, we get: $\nabla (x)_{{k{ + }1}}^{{\overline{F} }} = (x)_{k}^{{\overline{F} }} - (x)^{ - } = (x)_{{k{ + }1}}^{{\overline{F} }}$,$\nabla (x)_{{k{ + }1}}^{{\overline{F} }}$ and $(x)_{k}^{{\overline{F} }}$ meet $\nabla (x)_{{k{ + }1}}^{{\overline{F} }} \subseteq (x)_{k}^{{\overline{F} }}$, or $\nabla (x)_{k + 1}^{{\overline{F} }} \cup (x)_{k}^{{\overline{F} }} \ne \emptyset$, we get (27); or $\alpha^{F}$-microdata $\nabla (x)_{k + 1}^{{\overline{F} }}$ is intelligently decomposed-discovered from $\alpha^{{\overline{F} }}$-data block $(x)_{k}^{{\overline{F} }}$.

2. Because $card(\nabla (x)_{k + 1}^{{\overline{F} }} < card((x)_{k}^{{\overline{F} }}$ so $\eta_{k + 1}^{{\overline{F} }} = card(\nabla (x)_{k + 1}^{{\overline{F} }} /card((x)_{k}^{{\overline{F} }} ) < card((x)_{k}^{{\overline{F} }} /card(x)_{k}^{{\overline{F} }} ) = \eta = 1$, or $\eta_{k + 1}^{{\overline{F} }} - 1 < 0$, and we get (28). □

### Theorem 8

($\alpha^{{\overline{F} }}$-microdata $\Delta (x)_{k + 1}^{F}$ intelligent decomposition-discovery Theorem) Under the condition of reasoning (25), $\alpha^{{\overline{F}}}$-microdata $\Delta (x)^{F}$ is intelligently decomposed—discovered outside $\alpha^{{\overline{F} }}$-data block $(x)_{k}^{{\overline{F} }}$, or.

1. $$\Delta (x)_{k + 1}^{F}$$ and $$(x)_{k}^{F}$$ satisfy

29 $$\Delta (x)_{k + 1}^{F} \cap (x)_{k}^{F} \ne \phi$$

2. The separation coefficient $\eta_{k + 1}^{F}$ of $\Delta (x)_{k + 1}^{F}$ separated outside $(x)_{k}^{F}$ satisfies

30 $$\eta_{k + 1}^{F} - 1 > 0$$

where $$\eta_{k + 1}^{F} = card(\Delta (x)_{k + 1}^{F} )/card((x)_{k}^{F} )$$$$,1 = card(x)_{k}^{F} /card((x)_{k}^{F} ) = \eta = 1$$ is the Self separation coefficient of $$(x)_{k}^{F}$$.

Its proof is similar to Theorem 7, and will be omitted. The Propositions 5 is a direct conclusion of Theorems 7 and 8.

### Propositions 5

Under the condition of reasoning (26), $\alpha^{F}$-microdata $\nabla (x)_{k + 1}^{{\overline{F} }}$ and $\alpha^{{\overline{F} }}$-microdata $\Delta (x)_{k + 1}^{F}$ are intelligently decomposed—discovered within $(x)_{k}^{{\overline{F} }}$ and outside $(x)_{k}^{F}$ respectively.

## Microdata intelligent-security acquisition algorithm

**Figure 2:**
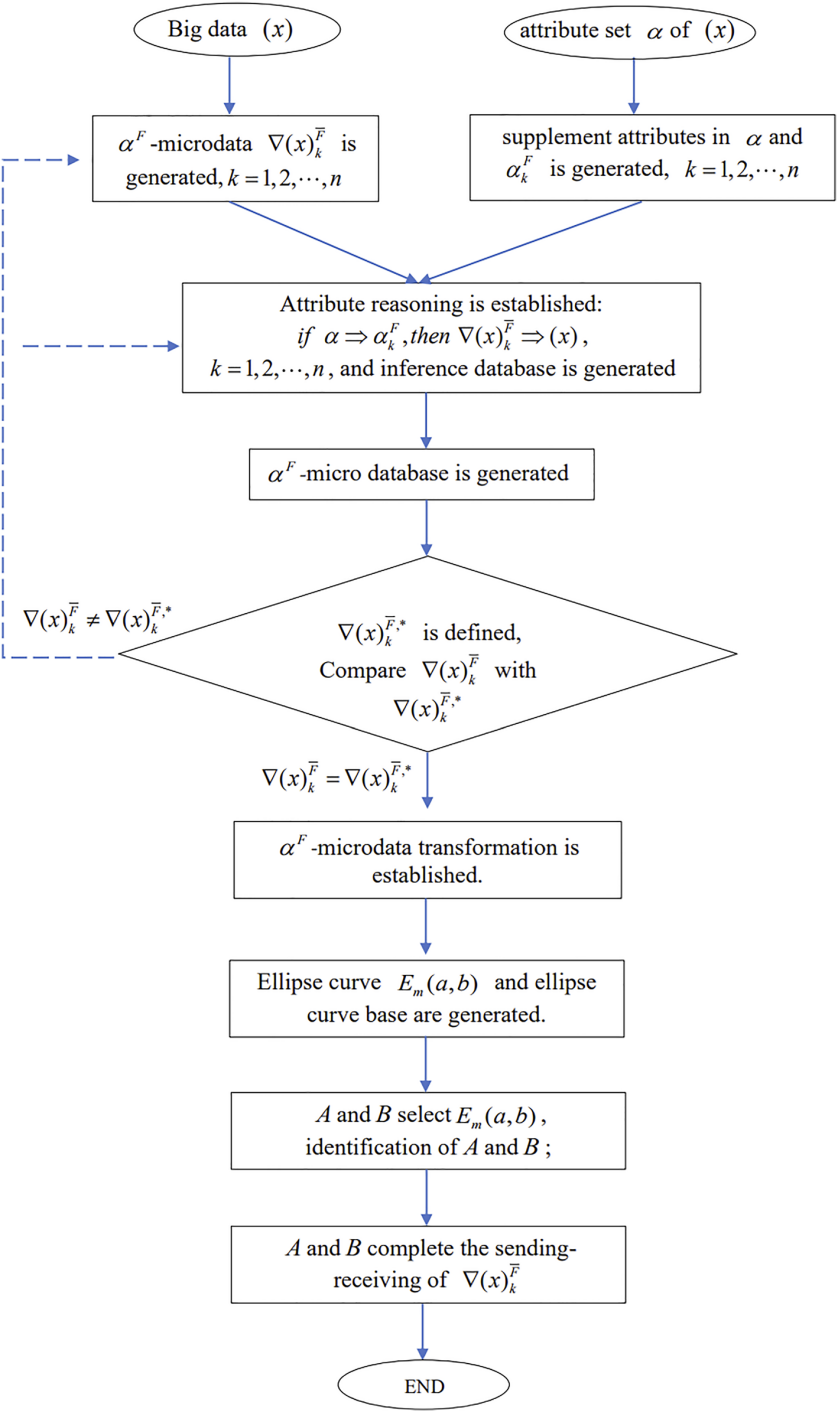
$\alpha^{F}$-microdata intelligent-security acquisition algorithm.

In this section, only the intelligent decomposition and secure acquisition algorithms of $\alpha^{F}$**-**microdata are given, it is a part of $(\alpha^{F} ,\alpha^{{\overline{F} }} )$-microdata intelligent decomposition and secure acquisition algorithm; the complete $(\alpha^{F} ,\alpha^{{\overline{F} }} )$-microdata intelligent decomposition and secure acquisition algorithm is omitted. The $\alpha^{F}$-microdata intelligent decomposition—security acquisition algorithm is shown in Fig. 2.

The detailed process of the algorithm is as follows:

(1) Big data $(x)$ and its attribute set $\alpha$ are given, which are the initial values of the algorithm;

(2) $\alpha^{F}$-microdata $\nabla (x)_{k}^{{\overline{F} }}$ is generated; supplement attributes in $\alpha$ and $\alpha_{k}^{F}$ is generated, $k = 1,2,\ldots,n$;

(3) Attribute reasoning is established:$if \, \alpha \Rightarrow \alpha_{k}^{F} ,then \, \nabla (x)_{k}^{{\overline{F} }} \Rightarrow (x)$, $k = 1,2,\ldots,n$, and inference database is generated;

(4) $\alpha^{F}$-micro database is generated;

(5) Given the standard microdata $\nabla (x)_{k}^{{\overline{F} ,*}}$, Compare $\nabla (x)_{k}^{{\overline{F} }}$ with $\nabla (x)_{k}^{{\overline{F} ,*}}$, if $\nabla (x)_{k}^{{\overline{F} }} \ne \nabla (x)_{k}^{{\overline{F} ,*}}$, then return to (2) and (3) and start cycle; if $\nabla (x)_{k}^{{\overline{F} }} = \nabla (x)_{k}^{{\overline{F} ,*}}$, then go to the next step;

(6) $\alpha^{F}$-microdata transformation is established;

(7) Ellipse curve $E_{m} (a,b)$ and ellipse curve base are generated;

(8) $A$ and $B$ select $E_{m} (a,b)$, identification of $A$ and $B$;

(9) $A$ and $B$ complete the sending-receiving of $\nabla (x)_{k}^{{\overline{F} }}$;

(10) The algorithm ends.

By using the preliminary concept in "Dynamic model with attribute conjunction" section, the theoretical results are deduced from "Data block decomposition and microdata generation" section and "Attribute reasoning and intelligent discovery of microdata" section and the intelligent security acquisition algorithm in "Microdata intelligent-security acquisition algorithm" section, and we get "Microdata encryption–decryption and its secure acquisition-application" section.

## Microdata encryption–decryption and its secure acquisition-application

The example in this section comes from $\alpha^{F}$-data block $(x)_{t}^{{\overline{F} }}$ of “commodity big data $(x)$”, $t = 1,2,\ldots,n$;the name of $(x)_{t}^{{\overline{F} }}$ is **“**commodity profit estimation”;$x_{i} \in (x)_{t}^{{\overline{F} }}$ is the commodity, $\alpha_{t}^{F}$ is the attribute set of $(x)_{t}^{{\overline{F} }}$, and $\alpha_{i} \in \alpha_{t}^{F}$ is the attribute of $x_{i}$; for convenience, the names of $x_{i}$ and $\alpha_{i}$ are omitted.

31 $$(x)_{t}^{{\overline{F} }} = \{ x_{1} ,x_{2} ,x_{3} ,x_{4} ,x_{5} ,x_{6} ,x_{7} ,x_{8} \}$$

32 $$\alpha_{t}^{F} = \{ \alpha_{1} ,\alpha_{2} ,\alpha_{3} \}$$

$\alpha_{i} = \alpha_{1} \wedge \alpha_{2} \wedge \alpha_{3}$ is the attribute of $x_{i}$,$y_{1} \sim\;y_{8}$ is the sales market of $x_{1} \sim\;x_{8}$, and $y_{1} \sim\;y_{8}$ is distributed in $8$ areas of the city.

### Profit Estimation of Commodity $x_{i}$

Make $\lambda$ surveys on the profit value of $x_{i}$, and use the statistical methods to get Table 1.

**Table 1:** $\xi_{i}$ is the pre estimate of $x_{i}$ profit,$i = 1,2,\ldots,8$.

$x_{i}$	$x_{1}$	$x_{2}$	$x_{3}$	$x_{4}$	$x_{5}$	$x_{6}$	$x_{7}$	$x_{8}$
$\xi_{i}$	7	—	—	9	10	—	—	9

The "—" in Table 1 indicates that the profit valuation $\xi_{i}$ is between 2 and 3;$\xi_{1} = 7$, $\xi_{4} { = 9}$, $\xi_{5} = 10$ and $\xi_{8} { = 9}$ are $7{\text{\% }}$, $9{\text{\% }}$, $10{\text{\% }}$ and $9{\text{\% }}$ respectively. The commodity profit investigator obtains two new attributes $\alpha_{4}$ and $\alpha_{5}$ of $x_{1}$, $x_{4}$, $x_{5}$ and $x_{8}$ while obtaining $\xi_{i}$, where $\alpha_{4}$ stands for that $x_{1}$, $x_{4}$, $x_{5}$ and $x_{8}$ installments are allowed; $\alpha_{5}$ stands for that $x_{1}$, $x_{4}$, $x_{5}$ and $x_{8}$ allowing return or exchange after purchase. When both $\alpha_{4}$ and $\alpha_{5}$ exist, $\alpha_{t}^{F}$ in (32) generates $\alpha_{{{\text{t}} + 1}}^{F}$.

33 $$\alpha_{t + 1}^{F} = \alpha_{t}^{F} \cup \{ \alpha_{4} ,\alpha_{5} \} = \{ \alpha_{1} ,\alpha_{2} ,\alpha_{3} ,\alpha_{4} ,\alpha_{5} \}$$

### Intelligent Generation of $\alpha_{t + 1}^{F}$-microdata $\nabla (x)_{t + 1}^{{\overline{F} }}$

By using (24) in "Attribute reasoning and intelligent discovery of microdata" section, we get:

34 $$if\,\alpha_{t}^{F} \Rightarrow \alpha_{t + 1}^{F} ,then\,\,\nabla (x)_{t + 1}^{{\overline{F} }} \Rightarrow (x)_{t}^{{\overline{F} }}$$

(34) gives

$$\begin{aligned} \nabla (x)_{t + 1}^{{\overline{F}}} & = (x)_{t}^{{\overline{F}}} - \{ x_{1} ,x_{3} ,x_{6} ,x_{7} \} \\ & = \{ x_{1} ,x_{4} ,x_{5} ,x_{8} \} \\ \end{aligned}$$

The attribute of $\forall x_{i} \in (x)_{t + 1}^{{\overline{F} }}$ is $\alpha_{i} = (\alpha_{1} \wedge \alpha_{2} \wedge \alpha_{3} )\wedge \alpha_{4} \wedge \alpha_{5}$, $i = 1,4,5,8$.

By using Table 1, the profit values composition points $(\xi_{1} ,\xi_{4} ) = (7,9)$ and $(\xi_{5} ,\xi_{8} ) = (10,9)$ of $x_{1}$, $x_{4}$, $x_{5}$ and $x_{8}$ are obtained.

### Secure acquisition application of $\alpha_{k + 1}^{F}$-microdata $\nabla (x)_{k + 1}^{{\overline{F} }}$

**Ellipse curve model and its encryption decryption algorithm**

Call $E(k) = \left\{ {(x,y)\left| {x,y \in k,p(x,y) = 0} \right.} \right\} \cup \left\{ {\mathcal{O}} \right\}$ is an ellipse curve, which is established by the following equation proposed by Koblitz^27^:

35 $$y^{2} = x^{3} + ax + b$$

where discriminant $D = (4a^{3} + 27b^{2} )\bmod m \ne 0$,$k$ is a number field, $a,b \in k$;${\mathcal{O}}$ is a point at infinity, $m$ is a prime number. Equation (35) is a simplified form of weisrstrass equations $y^{2} + a_{1} xy + a_{3} y = x^{3} + a_{2} x^{2} + a_{4} x + a_{6}$.

Taken $P(x_{1} ,y_{1} ),Q(x_{1} ,y_{1} ) \in E(k)$ arbitrarily,$R(x_{3} ,y_{3} )$ are points generated by $P(x_{1} ,y_{1} ) \oplus Q(x_{1} ,y_{1} )$ in $E(k)$ arbitrarily, $\oplus$ is the dot add operation in $E(k)$,$R(x_{3} ,y_{3} )$ is obtained as follows:

1. If $P(x_{1} ,y_{1} ) \ne Q(x_{2} ,y_{2} )$, then there are
  $$x_{3} = (\lambda^{2} - x_{1} - x_{2} )\bmod m,$$
  36 $$y_{3} = (\lambda (x_{1} - x_{3} ) - y_{1} )\bmod m,$$
  $$\lambda = \frac{{y_{2} - y_{1} }}{{x_{2} - x_{1} }}.$$
2. If $P(x_{1} ,y_{1} ) = Q(x_{2} ,y_{2} )$, then there are
  $$x_{3} = (\lambda^{2} - x_{1} - x_{2} )\bmod m$$
  37 $$y_{3} = (\lambda (x_{1} - x_{3} ) - y_{1} )\bmod m,$$
  $$\lambda = \frac{{3x_{1}^{2} + a}}{{2y_{1} }}.$$

Let *P*_*m*_ be the plaintext of $\alpha_{k + 1}^{F}$-microdata $\nabla (x)_{k + 1}^{{\overline{F}}}$, and $C_{m}$ be the ciphertext of $P_{m}$. *A* is the encryptor of plaintext *P*_*m*_ and the sender of ciphertext *C*_*m*_; *B* is the decryptor of ciphertext *C*_*m*_ and the accepter of plaintext $P_{m}$.$n_{A}$ is the private key to *A*, $P_{A} = n_{A} G$ is the public key of *A*; $n_{B}$ is the private key to $B$,$P_{B} = n_{B} G$ is the public key of $B$;$P_{A}$ is given to $B$ publicly and $P_{B}$ is given to $A$ publicly. $A$ and $B$ choose the same basic point $G \in E(k)$,$n_{A} ,n_{B} \in N^{ + }$.

I Information encryption process: $A$ chooses $P_{m} = \nabla (x)_{k + 1}^{{\overline{F} }} ,k \in N^{ + }$, the public key of $A$ and $B$ is $P_{B}$, then $A$ gives the ciphertext $C_{m}$ of $P_{m}$ as follows:

38 $$C_{m} = \{ kG,P_{m} + kP_{B} \}$$

Then $A$ sends $C_{m}$ to $B$.

II Information decryption process: $B$ accepts $C_{m}$, if $n_{B} (kG) = C_{m}^{1}$,$P_{m} + kP_{B} = C_{m}^{2}$, then $B$ obtains plaintext $P_{m}$ from ciphertext $C_{m}$

39 $$\begin{aligned} C_{m}^{2} - C_{m}^{1} & = P_{m} + kP_{B} - n_{B} (kG) \\ & = P_{m} + kP_{B} - k(n_{B} G) \\ & = P_{m} + kP_{B} - kP_{B} = P_{m} \\ \end{aligned}$$

where $P_{m}$ is the point composed of profit values $\xi_{1} ,\xi_{4} ,\xi_{5}$ and $\xi_{8}$ of $x_{1}$, $x_{4}$, $x_{5}$, and $x_{8}$: $(\xi_{1} ,\xi_{4} ) = (7,9)$,$(\xi_{5} ,\xi_{8} ) = (10,9)$.

Using $(\xi_{1} ,\xi_{4} ),(\xi_{5} ,\xi_{8} )$ to determine ellipse curve $y^{2} = x^{3} + ax + b\bmod m = x^{3} + x + 6\bmod 11$, Get point set: $E_{m} (a,b) = \{ (2,4),(2,7),(3,5),(3,6),(5,2),(5,9),(7,2),(7,9),(8,3),(8,8),(10,2),(10,9)\}$; obviously: $(\xi_{1} ,\xi_{4} ) = (7,9)$ and $(\xi_{5} ,\xi_{8} ) = (10,9) \in E_{m} (a,b)$.

$A$ chooses $k = 3$, the basic point is $G = (2,7)$,$n_{B} = 7$, $P_{B} = n_{B} G$ is the private key and public key of *B* respectively.

1. *A* chooses $P_{m} = (7,9) = 8G$, assume $k = 3$ and the public key of $G$ and $B$ is $P_{B}$,$A$ gives the ciphertext $C_{m}$ of $P_{m}$:

40 $$\begin{aligned} C_{m} = \{ kG,P_{m} + kP_{B} \} & = \{ 6G,8G + 3(7 \times 2G)\} \\ & = \{ 6G,8G + 42G\} = \{ 6G,50G\} \\ & = \{ 6G,2G\} = \{ (5,9),(2,7)\} \\ \end{aligned}$$

2. After $B$ accepts $C_{m}$, use $n_{B}$ to get $P_{m}$

41 $$\begin{aligned} C_{m}^{2} - C_{m}^{1} & = P_{m} + kP_{B} - n_{B} (kG) \\ & = 2G - 7(3G) = 2G - 42G \\ & = - 40G = - 4G = 8G = (7,9) = P_{m} \\ \end{aligned}$$

The encryption–decryption of $P_{m} = (10,9) = 12G$ is similar to (40) and (41), and which will be omitted.

**Important description of application examples**

1. The application example of the paper is taken from the "commodity profit estimation" block in "commodity big data", which is the compression and simplification of the original case. Because of the special nature of the example, some information can not be made public. The profit value $\xi_{i}$ of commodity $x_{i}$ is an important secret of company $C$; the profit value $\xi_{i}$ should be safe, should not be disclosed, and should not be stolen by others. Company $C$ shall safely receive the $\xi_{i}$ which sent by market investigators and shall make the decision on commodity $x_{i}$ access to the market.

2. In the competition of commodity market, if the profit value $\xi_{i}$ of $x_{i}$ is stolen by others, then company $C^{*}$, which produces similar commodity $x_{i}$, will quickly occupies the market, thus company $C$ will lose the market and the profit of the commodity. Therefore, the security of data transmission is particularly important, the encryption and decryption process in the information security algorithm ensures the security transmission of information.

3. The security of ellipse curve cryptography comes from the difficulty of solving discrete logarithms, point set $E_{m} (k)$ and dot add operation $\oplus$ defined on $E_{m} (k)$ form Abel Group $< E_{m} (k), \oplus >$. In the example, we only use $n_{B}$ and $P_{B}$ of $B$, and the $n_{A}$ and $P_{A}$ of $A$ are omitted.

**Confirmation of application examples**

The applications and the methods of the examples have been applied and confirmed in 8 business districts in Zhengzhou, China. The profits $\xi_{2} ,\xi_{3} ,\xi_{6}$ and $\xi_{7}$ of $x_{2} ,x_{3} ,x_{6}$ and $x_{7}$ in Table 1 are between 2 and 3; $y_{2} ,y_{3} ,y_{6}$ and $y_{7}$ were closed by company C in July 2020.

## Discussion

Big data is a branch of theory and application research with mathematical concept connotation, which has infiltrated into many application researches and aroused people's interest. What is the structure and characteristics of big data? Can it be classified? What is the basis for classification? In the existing literature, these problems have not been discussed. In this paper, the theory and application of Class I big data are presented by using the p-sets mathematical model with dynamic characteristics and attribute “Conjunction” characteristics. Restore the mathematical characteristics of big data with mathematical methods and models, and understand the mathematical structure and logical characteristics of big data. These characteristics are hidden in big data and do not attract people's attention. Finally, the paper selects the "financial security" data block in the "financial big data" as an example, uses the big data block theory given in this paper, and combines the elliptic curve to give the security algorithm of financial information encryption and decryption.

In the research of big data, most scholars combine mathematical models with big data to carry out application research, rarely pay attention to the theoretical research of big data, who do not recognize the dynamic and logical characteristics of big data, which lack strict reasoning and proof. In this paper, the author not only gives the theoretical research of big data by using mathematical models, but also pays attention to the combination of theoretical research with application research. Perhaps the research and results presented in this paper provide a research idea and mathematical method for researchers who are engaged in the fundamental theory and application of big data. The infiltration of mathematical concepts into the study of big data may make people acquire new theoretical understanding of big data, and it becomes an inevitable process from the simple study application to the theoretical study of big data.

In the inverse P-sets, attribute $\alpha_{j}$ of element $x_{j}$ conforms to the attribute “disjunctive” feature. Inverse P-sets model shares the same characteristics of as class II big data, which is a mathematical model and method to study class II big data. Applying inverse P-sets to study the characteristics and application of class II big data is the aim which we will meet in the future. Applying for inverse P-sets, The application and characteristics of class II big data is the aim which we will put forward in the future.

Function P-sets and function inverse P-sets are mathematical models and methods to study class I big data in function form and class II big data in function form respectively. All of P-sets, inverse P-sets, function P-sets, function inverse P-sets are the preparation for studying the application of big data, understanding the characteristics of big data, and giving mathematical models as well as methods.

## Data Availability

The datasets used and analysed during the current study available from the corresponding author on reasonable request.
